# In Remembrance: Reinhart Heinrich 1946–2006

**DOI:** 10.1371/journal.pcbi.0030018

**Published:** 2007-01-26

**Authors:** Thomas Höfer

Reinhart Heinrich, former professor at Humboldt University, Berlin, and best-known as a founder of metabolic control theory, died October 23, 2006, at the age of 60. Among his many services to the scientific community, Reinhart was associate editor of *PLoS Computational Biology*. His far-reaching theoretical work on metabolism, signal transduction, and other cellular processes has made him one of the most influential forerunners of present-day systems biology.

Having been educated as a theoretical physicist at Technical University, Dresden, Reinhart conducted his postdoctoral research in the early 1970s at the Institute of Biochemistry in East Berlin. He could not fail to notice the absence of mathematical theory from cell biology as compared with other natural sciences. Enzyme kinetics was a notable exception. However, how enzymes affect the flux through a metabolic pathway was still discussed using the rather vague term *rate-limiting step.* Working with Tom Rapoport on mathematical models of glycolysis in red blood cells, Reinhart discovered a precise and general definition of rate limitation in metabolic pathways. The parallel development of metabolic control theory by Henrik Kacser and Jim Burns in Edinburgh shows that the time was ripe for a quantitative understanding of metabolic regulation. Instead of postulating a single rate-limiting step, Reinhart's and Henrik Kacser's theories evaluated the *degree* of flux control exerted by an individual enzyme in a linear pathway or in a more complex network. The corresponding measure—termed flux control coefficient—turned out to be a truly systemic quantity, depending not only on the kinetic parameters of the enzyme itself but also on those of other enzymes, as well as on the position of the reaction in the network. Several years after its original publication, metabolic control theory became widely absorbed by biochemists. Control coefficients have been measured for many pathways, confirming the theoretical prediction that flux control is frequently shared by several reactions. This finding has recently become of very practical importance for the genetic engineering of large metabolic networks in biotechnology.

The dual approach—modeling concrete cellular processes and, at the same time, searching for general laws—has been a characteristic of Reinhart's work. The areas he worked in were amazingly diverse, including metabolic control, osmoregulation, cell shapes, signal transduction, vesicular transport, protein translation and transport, as well as the population dynamics of malaria parasites.

Perhaps his most favored questions were those of evolution. To understand the kinetic design of enzymes and enzymatic reaction networks, Reinhart strove to rationalize, in mathematical terms, the selective pressures and physico–chemical constraints that these systems were subjected to. Reinhart's work on this topic is full of original insight and makes specific predictions, some of which have begun to be tested successfully in recent years.

Reinhart was author of more than 150 research articles and, together with Stefan Schuster, wrote the book *The Regulation of Cellular Systems*—already a classic of cell systems biology. In addition to this large body of original work, he was a gifted mentor of young scientists and for more than ten years ran the highly successful interdisciplinary graduate program “Dynamics and Evolution of Cellular Processes” at Humboldt University, Berlin. Reinhart's many talents made him appear as a modern Renaissance man. He played the violin, wrote poetry, and published an autobiographic novel *(Jenseits von Babel).* Reinhart had a large international circle of friends and collaborators by whom he will be remembered for his kindness, generosity, and contagious humor.

***From the Editor:*** *Reinhart was a recent, highly commended, member of the* PLoS Computational Biology *Editorial Team. Thomas has made it abundantly clear why we asked Reinhart to join us. He was an active and timely contributor and he will be sorely missed by all of us at PLoS as well as by the rest of the scientific community. Our condolences go out to his family.* *— Philip E. Bourne, Editor-in-Chief*

## 

**Reinhart Heinrich at the reception in honor of his 60th birthday in April 2006:**
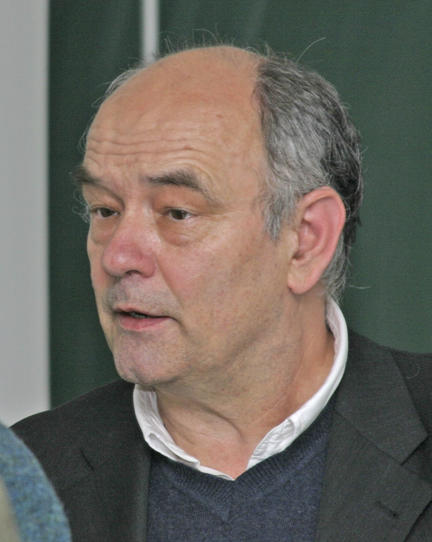
(Photo by Jana Schütze)

